# Primary testicular necrotizing vasculitis clinically presented as neoplasm of the testicle: a case report

**DOI:** 10.1186/1477-7819-9-63

**Published:** 2011-06-14

**Authors:** Anton Maričić, Sanja Štifter, Maksim Valenčić, Gordana Ðorđević, Dean Markić, Josip Španjol, Stanislav Sotošek, Željko Fučkar

**Affiliations:** 1 Department of Urology, University Hospital Rijeka, Rijeka, Croatia; 2 Department of Pathology, School of Medicine, University of Rijeka, Rijeka, Croatia

**Keywords:** Necrotizing vasculitis, testicular neoplasm, radical orchiectomy, ultrasound

## Abstract

We present a case of necrotizing vasculitis with the testicle as the isolated affected organ. A 25-year-old man, pretreated for epididymo-orchitis, presented with a presumed testicular neoplasm. Radical orchiectomy was performed and diagnosis of necrotizing vasculitis was established. In the absence of any other sign of systemic disease, the diagnosis of isolated necrotizing vasculitis of the testis was confirmed. Two years after the operation, the patient showed no symptoms of systemic disease.

## Background

Symptomatic vasculitis confined to the testis without clinical or laboratory evidence of systemic disease is not a common finding [1-10]. It is difficult to diagnose this condition clinically or using noninvasive methods. Therapy for this condition remains controversial. We describe a case with an unusual presentation simulating a testicular neoplasm.

### Case presentation

A 25-year-old Caucasian man went to a general practitioner because of right testicular swelling and was treated with oral antibiotics for presumed epididymo-orchitis. Over the next 10 days, swelling increased, the testis became painful, body temperature increased to 38°C, and the patient was referred for urological assessment. The patient was admitted to the hospital for parenteral therapy, because peroral antibiotic therapy (ciprofloxacin) was not effective.

**Figure 1:**
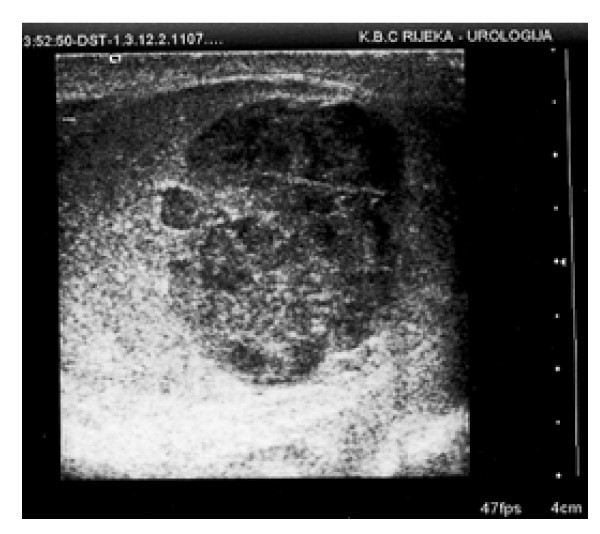
**Ultrasound: hypoechogenic focal lesion in the lower pole of right testicle**.

Upon physical examination, the right testicle was enlarged and painful on palpation, and the skin of the right hemiscrotal region was red and warm. Pain increased gradually and worsened slightly with time, but this type of pain was not typical of the presumed diagnosis. A palpable mass was found in the lower part of the testicle. Structures of the funiculus were painless. Prehn's sign was negative. Laboratory findings demonstrated a leukocytes count of 11.4 × 10^9^/L, an erythrocyte sedimentation rate of 30 mm/h, C reactive protein (CRP) level of 61.7 mg/L and normal serum tumor marker levels. The results of a full blood count, serum electrolyte measurements and liver function tests were normal as were chest radiography findings. Urinalysis and culture results were negative. The initial clinical diagnosis was epididymo-orchitis, and parenteral antibiotic therapy was started (combined amoxicillin/clavulanic acid 1.2 g three times daily and gentamicin 160 mg once daily). On the second day of hospitalization, the patient became afebrile, but after 10 days of therapy, no improvement was observed. Scrotal ultrasound examination revealed an abnormal right testis with a focal lesion (2.5 × 2 cm) in the lower part. The lesion was hypoechogenic compared with the surrounding testicular tissue, and suggested the existence of a tumor mass (Figure 1). Left testis and epididymis were sonographically normal. Doppler ultrasound examination revealed well vascularized right and left testes. The focal lesion of the right testis was also vascularized, similar to the surrounding normal testicular parenchyma. This finding practically excluded testicular torsion, segmental testicular infarction and orchitis as possible diagnoses. Because ultrasound findings of the right testicle were highly indicative of testicular neoplasm, right radical orchiectomy was performed via an inguinal incision.

#### Histopathological findings

**Figure 2:**
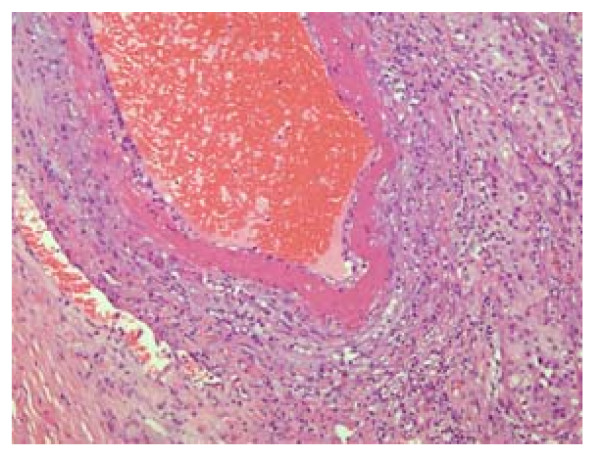
**Hematoxylin eosin (HE) staining showing the medium-sized artery in the testicular parenchyma showing fibrinoid necrosis and segmental involvement of moderate inflammatory cell infiltrate and perivascular inflammation (200x)**.

**Figure 3:**
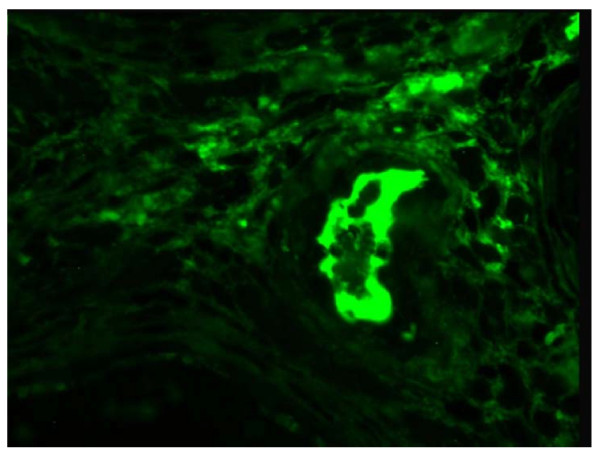
**Immunofluorescence staining detected deposits of fibrin in testicular vessels affected with fibrinoid necrosis**. This represents a morphological hallmark of necrotizing vasculitis (200x).

The testicle measured 4 × 3.5 × 2.5 cm and contained well-demarcated areas of hemorrhage, 3 cm in diameter. The epididymis, investing membranes and spermatic cord appeared grossly normal. Microscopy showed the presence of a patchy, necrotizing vasculitis affecting medium-sized and small-sized arteries of the testicle (Figure 2). Several vessels showed fibrinoid necrosis of their walls and muscular layer detachment with or without a transmural infiltrate composed of polymorphonuclear leukocytes and lymphocytes. Immunofluorescence staining for fibrin was also performed, and positive fibrin deposits were identified in arterial walls affected with fibrinoid necrosis (Figure 3).

#### Follow-up

The postoperative course was uneventful. After the operation, extensive clinical evaluation was performed to exclude other systemic diseases characterized by vasculitis. This included: complete blood count; erythrocyte sedimentation rate; CRP; urinalysis; immunoglobulin serum level; immunological blood tests, such as rheumatoid factor, antinuclear antibody tests and anti-neutrophil cytoplasmic autoantibody test; complement tests; human leukocyte antigen tissue typing tests; ultrasound of the abdomen; endoscopic examination of the ear, nose and throat; chest X-ray; and ophthalmologist examination. All test results were normal. There was no sign of systemic disease. Two years after the diagnosis, systemic disease had not developed.

## Discussion

The most common appearance of testicular vasculitis is as part of a multiorgan or systemic disease. Involvement of the testicles is seen less frequently in Wegener's granulomatosis, Henoch-Schönlein purpura, giant cell arteritis, and rheumatoid arthritis, whereas testicle involvement is commonly associated with polyarteritis nodosa [3-5]. The microscopically observed changes are almost identical in all vasculitis seen in other systemic disorders. The results of postmortem studies suggest that the testis is involved in 38-86% of cases of polyarteritis nodosa. At the same time, less than 18% of these cases are symptomatic, and most will show other manifestations of polyarteritis nodosa [6,7].

Isolated testicular vasculitis is not a common condition [10-13]. It is usually found in young people, as in our patient [12]. From the present literature findings, it remains unclear whether such cases represent truly isolated vasculitis or solely an unusual primary presentation site. The pathogenesis of isolated organ vasculitis is unknown, as is why only one organ may be affected. Additionally, it is unknown whether such cases carry the risk of subsequent progression, and if so, the risk remains to be determined. It is not known whether isolated vasculitis has a better prognosis than does systemic disease. This is important because the classic form of polyarteritis nodosa carries significant risks of mortality and morbidity, even with treatment, and has a high rate of relapse [9].

The conditions presenting as pain in the testicle or epididymitis have been previously reported, but presentation with clinical features suggestive of testicular neoplasm is even more exceptional [7,10]. In the majority of reported cases, clinical or laboratory evidence of disease in other organ systems on presentation was present or developed subsequently within a short time period [5].

Testicular necrotizing vasculitis is impossible to diagnose without tissue analysis. Our patient first presented with symptoms and signs in favor of inflammation (orchitis as concomitant disease cannot be excluded). However, when ultrasound with Doppler was performed, it was obvious that inflammation was not the cause of this lesion. Additionally, because the lesion was well vascularized, testicular torsion and segmental testicular infarction were excluded. Testicular neoplasm remained the most probable diagnosis. After orchiectomy, histopathological findings were used to investigate the existence of necrotizing vasculitis. Histopathological characteristics observed in necrotizing vasculitis are mainly restricted to blood vessels. Fibrinoid necrosis is the morphological hallmark of the disease. The walls of small and medium-sized testicular arteries are affected, as shown in this case report. Notably, hemorrhagic necrosis occurs in other pathological conditions, such as testicular torsion, infarction and inflammation.

Serological markers such as CRP and von Willebrand factor are possible indicators of endothelial injury in systemic vasculitis but may not reflect the activity in isolated organ disease. Ultrasound examination may fail to show any abnormality but can also demonstrate the existence of a hypoechogenic mass, as in our patient [12]. Magnetic resonance imaging is a more sensitive technique that can demonstrate focal testicular infarction, but, at present, the only "diagnostic tool" for vasculitis is histological confirmation.

To treat our patient with potentially toxic immunosuppressive therapy with the added risk of sterility, despite the lack of clinical and objective laboratory evidence of systemic disease, presents a difficult clinical dilemma. Waterfield et al. reported on isolated testicular vasculitis treated by immunosuppressive medications. Despite therapy, the remaining testis became affected one year later [10]. That patient responded well to an increase in immunosuppressive therapy. McGuirre et al. recommended close surveillance without additional therapy [12]. Because of his young age, we elected to perform close follow-up of our patient, instead of immunosuppressive therapy. Two years after diagnosis, the patient is still without symptoms of systemic disease. This is the longest asymptomatic period in a case of testicular vasculitis reported in the literature. In view of the high relapse rate associated with polyarteritis nodosa, long-term follow up for these patients is essential. However, the absence of serological markers of disease activity makes monitoring of any future relapse quite difficult.

## Conclusion

Primary testicular manifestation of necrotizing vasculitis is not a common finding. It is very important for pathologists and clinicians to know that such an entity can initially present as a testicular mass. Follow-up of these patients is recommended due to the risk of relapse; however, due to the rarity of the condition, the appropriate strategies for treatment and follow-up remain to be determined.

## Consent

Written informed consent was obtained from the patient for publication of this Case report and any accompanying images. A copy of the written consent is available for review by the Editor-in-Chief of this journal.

## Abbreviations

CRP: C reactive protein.

## Competing interests

The authors declare that they have no competing interests.

## Authors' contributions

MA, SS and MD tracked the clinical data and drafted the manuscript. FŽ, VM and ŠJ participated in the design of the study and coordination and helped to draft the manuscript. GÐ and SŠ provided the pathological technique.

All authors read and approved the final manuscript.
